# Surgically-induced brain injury: where are we now?

**DOI:** 10.1186/s41016-019-0181-8

**Published:** 2019-12-16

**Authors:** Zachary D. Travis, Prativa Sherchan, William K. Hayes, John H. Zhang

**Affiliations:** 1 Department of Earth and Biological Sciences, School of Medicine, Loma Linda University, Loma Linda, CA 92354 USA; 2 Department of Physiology and Pharmacology, School of Medicine, Loma Linda University, Loma Linda, CA 92354 USA; 3 Department of Anesthesiology, School of Medicine, Loma Linda University, Loma Linda, CA 92354 USA

**Keywords:** Brain injury, Cerebral edema, Preconditioning, Neurotherapeutics, Venom therapies

## Abstract

Neurosurgical procedures cause inevitable brain damage from the multitude of surgical manipulations utilized. Incisions, retraction, thermal damage from electrocautery, and intraoperative hemorrhage cause immediate and long-term brain injuries that are directly linked to neurosurgical operations, and these types of injuries, collectively, have been termed surgical brain injury (SBI). For the past decade, a model developed to study the underlying brain pathologies resulting from SBI has provided insight on cellular mechanisms and potential therapeutic targets. This model, as seen in a rat, mouse, and rabbit, mimics a neurosurgical operation and causes commonly encountered post-operative complications such as brain edema, neuroinflammation, and hemorrhage. In this review, we elaborate on SBI and its clinical impact, the SBI animal models and their clinical relevance, the importance of applying therapeutics before neurosurgical procedures (i.e., preconditioning), and the new direction of applying venom-derived proteins to attenuate SBI.

## Background

Damage to brain tissue occurs frequently at the periphery of a resection site. The delicate and intricate architecture of the brain presents severe challenges for neurosurgery; in fact, some specific neurosurgical operations where the brain stem, spinal cord, and posterior cranial vault are involved have been linked to post-operative neurological deficits, no matter how precise and careful the surgeon is [1–3].

After a craniotomy has been performed and the meninges resected, the brain is extremely susceptible to mechanical injury. Surgical brain injury (SBI) comprises a form of injury that inadvertently results from damaged brain tissue at the perisurgical site due to neurosurgical maneuvers such as incision, retraction, and electrocauterization, all of which are essential surgical techniques. Although, through modern science, there has been a decrease in the level of invasiveness with endoscopic surgeries and stereotaxic-guided procedures, coupled with an increase in the specificity of post-operative care, there remains unavoidable injury which negatively impacts the patient, their family, and the health care system in the short and long term [4].

Brain edema, neuroinflammation, cellular death, and hemorrhage are post-operative complications that develop (within hours and continue for days after injury) following neurosurgical procedures and may lead to further injury by triggering secondary pathways that ultimately lead to long-term complications and neurological deficits [5–10]. To date, complications arising from SBI are not explicitly treated and are left to heal on their own. Therapies which directly target SBI are lacking, leaving a gap in post-care treatment. SBI not only poses a risk to all patients who undergo brain surgery but also eliminates certain patients from specific surgical procedures which are deemed more risky.

Every year, 13.8 million patients around the globe require surgery due to traumatic brain injury (TBI), stroke-related conditions, tumors, hydrocephalus, and epilepsy [11]. Millions of these surgical cases are in low- and middle-income countries where acute care is hard to come by. It is imperative that we look toward a potential therapeutic which can diminish post-operative complications which may not only have a positive effect on the patient but also on low- and middle-income countries. Osmotic agents, diuretics, and steroids have been used to reduce the post-operative effects of these injuries and decrease the neurological deficits that may occur [12]. Steroids successfully attenuate tumorigenic edema, but in CRASH trials, steroids showed harmful effects after traumatic brain injury [13]. Currently, there are no standard treatment regimens to prevent the inevitable injuries associated with routine neurosurgical procedures [14].

Complications that lead to neurological deficits often result in a financial and legal quagmire. Patients and their families may suffer from devastating financial burdens. Physicians, and especially surgeons, have become all too familiar with a toxic medical-legal climate that has led to defensive medical practice by those in high-risk specialties. In a *Journal of the American Medical Association* study, nearly 75% of polled neurosurgeons confessed to avoiding particular procedures or high-risk patients out of the fear of malpractice suits being filed against them [15, 16]. Furthermore, a UK survey done by the Medical Defense Union stated that damage to underlying brain structures is the commonest complication for which patients successfully sue surgeons [17]. Even if there is no serious complication, neurosurgical patients have to be monitored closely, which translates into longer hospital stays and rising costs for the patient, healthcare system, and society. Diminishing perioperative risks may allow for an expansion of more aggressive surgical interventions and more patients being suitable for treatment.

The purpose of this review is to discuss the pathophysiology of SBI, animal models currently being used for investigation, and potential therapeutics that could provide neuroprotection for patients.

## Animal model for surgical brain injury

Animal models for brain injury allow investigators to study cellular signaling mechanisms by applying molecular techniques to the affected brain tissue. Upon successful determination of signaling pathways, key molecular targets for potential neuroprotection may be investigated [18]. First seen in 2006, Frontczak-Baniewicz et al. [19] demonstrated that an in vivo frontal temporal model could be used to study SBI. Jadhav et al. [14] created a replicable in vivo model which has been utilized for the past decade to study brain injury caused by neurosurgical procedures. This frontal lobe resection model is not intended to mimic any specific neurosurgery operation, rather it allows researchers to simulate a more general SBI by causing both cortical and parenchymal damage. This model produces a certain amount of brain tissue loss and injury that causes the neuronal death, blood-brain barrier (BBB) dysfunction, and brain edema that occur during routine neurosurgical operations. Previous reports have documented that the SBI animal model has localized brain edema and BBB disruption in the brain tissue surrounding the resection. The model allows researchers to study the post-operative complications of surgically induced brain injury, and the molecular mechanisms and signaling pathways involved, because it provides for consistently measurable edema via brain water content in the perilesional tissue. It also promotes measurement of neurological deficits following SBI, which is critical when investigating therapies for patients.

**Fig. 1:**
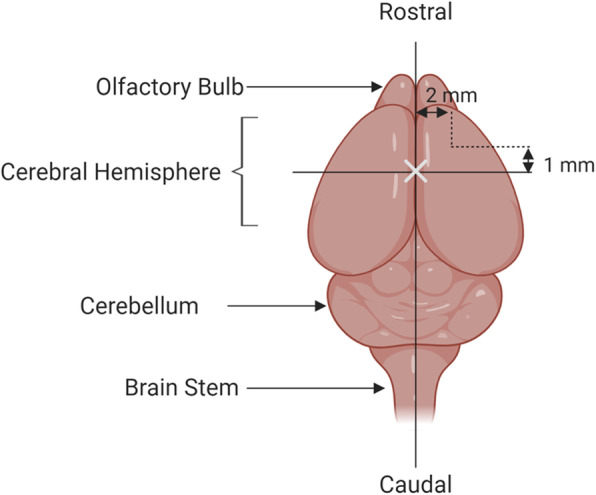
Partial right frontal lobectomy. Two incisions are made leading away from the bregma (X), 2-mm lateral and 1-mm proximal to the sagittal and coronal sutures

Investigators have adopted this model to rats, mice, and even rabbits. In brief, after anesthetizing the animal, the investigator exposes the frontal skull and then removes a bone flap to expose the right frontal lobe of the brain. After moving aside the dura, the investigator then makes two incisions to free the right frontal lobe from its surrounding tissue. Intraoperative packing and saline irrigation are used to control bleeding and induce hemostasis before placing the dura and skull cap back in their original position and suturing the skin (Fig. 1).

## Pathophysiology of surgical brain injury

**Fig. 2:**
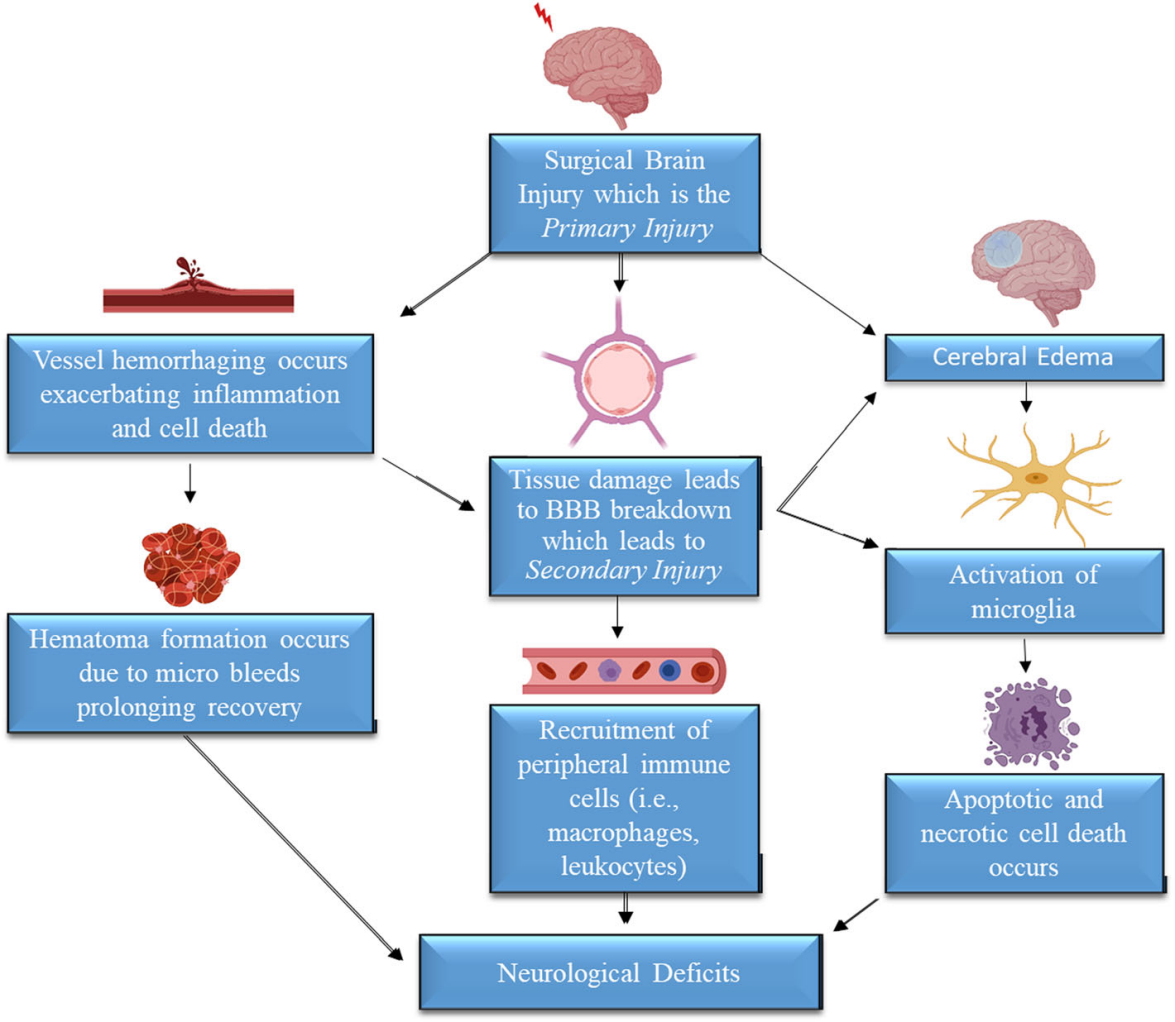
Primary and secondary injury due to SBI

As previously mentioned, SBI comprises a two-stage injury. Primary injury results from the mechanical forces during surgery, which are largely unavoidable, though minimally invasive techniques are increasingly utilized. Secondary injury arises from the cascade of cellular and metabolic processes put into motion because of the primary injury [20] (Fig. 2). This cellular cascade chiefly involves inflammatory molecules such as cytokines and prostaglandins. A key player in the propagation of the secondary injury is the breakdown of the blood-brain barrier (BBB).

The BBB is part of a complex and intricate barrier system which is tasked with maintaining homeostasis for the neural microenvironment [21, 22]. Three barriers actually exist between the blood and the central nervous system (CNS): the BBB, blood-cerebral spinal fluid (CSF) barrier, and the arachnoid barrier. The BBB is created by the endothelial cells that form the wall of the brain capillaries; the blood-CSF barrier is formed by the epithelial cells of the choroid plexus; and the arachnoid barrier is composed of the avascular arachnoid epithelium [21]. The BBB functions as a result of three properties: a physical barrier composed of tight junctions between cells reducing flux via the intercellular cleft or paracellular pathway; a transport barrier which mediates the movement of solutes; and a metabolic barrier [22]. All three properties of this collective barrier can be modulated either through homeostatic pathways or pathologies. Disruption to the BBB has been shown to increase post-operative brain edema and worsen neurological function. Trauma, for example, can generate bradykinin, a mediator of inflammation, which stimulates production and release of interleukin-6 (IL-6) from astrocytes, which in turn leads to opening of the BBB [23]. Without stable fluidity provided by the barrier, the CNS cannot function.

Four pathophysiological features of SBI merit special consideration: cerebral edema, neuroinflammation, cell death, and hemorrhage and will be explained in more depth. These pathologies are implicated in exacerbating the healing process for patients.

*Cerebral edema* is a common pathophysiological formation following surgery. Cerebral edema comprises excess accumulation of water in the intra- and/or extracellular spaces of the brain [24]. Cerebral edema results from a combination of endothelial cell damage, tight junction disruption, and abnormal transcellular transport [25]. Damage to cells and blood vessels triggers a multitude of cellular cascades, which amplifies injury. Calcium and sodium channels become activated, which causes a fluid imbalance and triggers cytotoxic processes. An inflammatory response is mounted, and microglial cells release free radicals and proteases which further the attack on cell membranes and capillaries [26]. The free radicals are toxic to cells, and macrophages, as well as activated microglial cells, form nitric oxide (NO), which is an additional source of free radicals. When the CNS is injured, mediators such as glutamate and extracellular potassium are released which causes swelling leading to damage of nerve cells [24].

Cerebral edema has been partitioned into three categories—vasogenic, cytotoxic, and interstitial edema. *Vasogenic cerebral edema* is the term used to describe the influx of fluid and solutes into the brain through an inadequate BBB, which primarily affects the white matter [27]. Vasogenic cerebral edema is the most common type of brain edema, and originates from the increased permeability of the capillary endothelial cells. The breakdown of the BBB allows for the movement of proteins and solutes that were originally in the intravascular space through the capillary wall into the extracellular space. *Cytotoxic edema* describes a cellular swelling that affects primarily the gray matter, and is seen in conditions such as head injury and hypoxia [28]. Cytotoxic edema is caused by swelling of glia, neurons, and endothelial cells, and begins within minutes after an insult [29]. *Interstitial edema* is known to occur in hydrocephalus patients and occurs when outflow of CSF is obstructed, leading to interventricular and eventually intracerebral pressure increase [30].

Brain edema leads to brain swelling. Clinical studies indicate that brain water content is a good indicator of brain swelling resulting from the edema. A 1% increase in brain water content is equivalent to a 4.3% increase in brain tissue volume [31, 32]. Rodent studies indicate that the brain water content of tissue surrounding the resection site increases by 3% or more during the first 72 h following surgery, and gradually resolves within a week after surgery [33–35]. As stated by the Monro-Kellie hypothesis, the totality of elements inside the skull is composed of the brain, CSF, and blood [36]. These three entities need to remain constant because of the skull’s rigidity. If, for example, there is excessive water formation, the brain and blood vessels surrounding the brain will be compressed. Cerebral edema leads to the expansion of brain volume against an enclosed skull and an increase in intracranial pressure (ICP). Elevated ICP can cause herniation, and can also decrease cerebral perfusion pressure, which promotes cerebral ischemia [36].

*Neuroinflammation* is a key player in the progression of brain edema after neurosurgical procedures. Previous SBI studies have successfully demonstrated that neuroinflammation is propagated through pro-inflammatory cytokines, activation of microglia, and infiltration of non-resident immune cells to the site of injury [8, 18, 37, 38]. Infiltrated peripheral immune cells release inflammatory mediators and promote oxidative stress and cell death, which contributes to progression of the injury [39, 40]. Identifying drug targets toward improving functional outcomes post-TBI requires a better understanding of neuroinflammation, including BBB dysfunction, activation of brain resident microglia and astrocytes, secretion of inflammatory mediators, and subsequent recruitment of peripheral immune cells [40–42]. When the BBB is disrupted, and the injured brain is infiltrated by peripherally derived immune cells (i.e., neutrophils and macrophages), resident astrocytes and microglia in the brain are activated.

*Cell death***,** specifically apoptotic and necrotic cell death, has been noted in SBI. Neuronal and glial cell deaths, as well as axonal injury, are the main contributors to the overall pathology of TBI [43]. Matchett et al. [33] demonstrated apoptotic neuronal death in an SBI model. Furthermore, Sulejczak et al., [10] demonstrated that neuronal apoptosis was accompanied by astrogliosis at the site of resection. In TBI models, apoptotic and necrotic neurons been identified not only at the site of injury post-trauma but also in regions remote from the site of injury days and weeks after trauma [26].

*Hemorrhage* is a critical issue in neurosurgery and is implicated in contributing to SBI [9, 44]. Firstly, intraoperative bleeding causes local ischemic insult and systemically plagues the cardiovascular system. While electrocauterization effectively controls bleeding and allows surgeons to be more invasive, healthy tissue becomes damaged by thermal injury. Secondly, SBI causes damage to the brain parenchyma, which damages the cerebral microvessels and leads to neurovascular unit pathophysiology. Disruption of the walls of the microvessels in the BBB activates the coagulation cascade. Since the integrity of the BBB becomes compromised after injury, the proteins thrombin, albumin, and fibrinogen can now enter the brain which causes neuroinflammation and apoptosis. Similar to cerebral edema, even a small increase in blood volume will cause the brain to herniate, leading to life-threatening complications.

Though hematoma formation can be mitigated through proper surgical management, its formation can contribute the propagation of neurological deficits [45]. Microbleeds that occur in the periphery may not be as detrimental as a bleed that is in the brain because of the limited space for expansion. Hematoma formation can increase pressure and force herniation to occur. On a cellular level, the presence of a hematoma is known to activate microglia and the complement cascade. As aforementioned, these two systems lead to an increase in inflammation and damage to healthy unaffected tissue. Limiting the amount of intraoperative bleeding may also reduce the size of a hematoma leading to more positive patient outcomes.

## Neurotherapeutics and preconditioning in surgical brain injury

Currently, clinical management of surgical brain injury is limited to nonspecific post-operative care (e.g., osmotherapy (mannitol, glycerol), diuretics, corticosteroids, and hyperventilation). Many promising therapeutic agents and strategies to mitigate complications of SBI have been evaluated experimentally in animal models (summarized in Table 1), with nearly all of these studies utilizing pre- or post-surgical treatments. Because of the electability of many neurosurgeries, with surgeries scheduled in advance, SBI presents a unique opportunity to test neuroprotection that may prove clinically relevant. In the following sections, we expand on the concept of preconditioning and propose the use of venom-derived proteins as a preconditioning therapy for SBI.

**Table 1:** Review of treatments and outcomes in studies using animal models for surgical brain injury

Reference	Model	Treatment	Outcomes
Matchett et al. 2006 [33]	Rat	Erythropoietin pre-treatment	Increased brain water content (BWC)
Lo et al. 2007 [46]	Mouse	NADPH oxidase knockout (KO) or apocynin pre-treatment	KO: increased neurological score (NS); apocynin: no effect
Jadhav et al. 2007 [35]	Rat	PP1 pre-treatment	Decreased BWC, vascular endothelial growth factor (VEGF), p-ERK1/2; increased zonula occludens-1 (ZO-1)
Yamaguchi et al. 2007 [34]	Rat	MMP inhibitor-1 preconditioning	Decreased BWC
Lee et al. 2008 [47]	Rat	Simvastin pre-treatment	No effect
Lee et al. 2008 [48]	Rat	Melatonin pre-treatment	5 and 15 mg/kg decreased lipid peroxidation (LPO), BWC; increased NS. 150 mg/kg decreased NS; increased BWC & LPO
Bravo et al. 2008 [49]	Mouse	L-histadine and thioperamide post-treatment	Increased BWC
Hyong et al. 2008 [7]	Rat	Rosiglitazone pre-treatment	Decreased myeloperoxidase (MPO) activity, Tumor Necrosis factor-α (TNF-α), Interleukin-1β (IL-1β)
Di et al. 2008 [50]	Rat	Aminoguanidine post-treatment	Decreased BWC, TNF-α, NF-κB; increased NS
Hao et al. 2009 [51]	Rat	Aminoguanidine post-treatment	Decreased malondialdehyde (MDA), aquaporin-4 (AQ-4); increased glutathione (GSH)
Jadhav et al. 2009 [52]	Mouse	Hyperbaric oxygen preconditioning	Decreased BWC, cyclooxgenaase-2 (COX-2), hypoxia-inducible factor-1α (HIF1A); increased NS
Westra et al. 2011 [53]	CD1 Mice	Hyperbaric (2.5 ATM) and normobaric oxygen (100% FiO_2_) pre-treatment	Decreased NS; increased BWC
Khatibi et al. 2011 [54]	CD57 Mice	Granulocyte-colony stimulating factor (GCSF) preconditioning and post-treatment	Preconditioning decreased cell death, BWC; post-treatment increased NS
Khatibi et al. 2011 [55]	CD57 Mice	Prostaglandin E2 EP1 receptor antagonist pre-treatment	No effect on BWC or cell death; increased NS
Jafarian et al. 2011 [56]	CD57 Mice	Myelin basic protein (MBP) pre-treatment	Preserved transforming growth factor beta-1 (TGFβ1); increased NS
Ayer et al. 2012 [8]	CD57 Mice	Myelin basic protein (MBP) pre-treatment	Decreased BWC, IL-1β; increased NS, TGFβ1
Eckermann et al. 2011 [57]	Rat	2.9 percent hydrogen concurrent with surgery	Decreased BWC; increased NS
Benggon et al. 2012 [58]	Rat	Dexmedetomidine pre-treatment	No effect
Manaenko et al. 2013 [59]	Rat	PAR-1 antagonist SCH79797 pre-treatment	Decreased BWC, cellular apoptosis
Zheng et al. 2014 [60]	Rabbit	Thymus tolerance pre-treatment	Decreased IL-1
Xu et al. 2014 [61]	Rat	Dexamethasone vs. progesterone pre-treatment	Progesterone decreased BWC and MMP-9 expression
Huang et al. 2014 [62]	Rat	Collagen-glycosaminoglycan matrix implantation	Increased VEGF, fibroblast growth factor-2 (FGF2), platelet-derived growth factor (PDGF)
Huang et al. 2015 [63]	Rat	PI3Kγ inhibitors AS252424 and AS605240 pre-treatment	Decreased BWC; increased NS
Huang et al. 2016 [64]	Rat	Valproic acid pre-treatment	Decreased BWC; no MMP inhibition; no effect on NS
Komanapalli et al. 2016 [65]	Rat	Epsilon aminocaproic acid pre-treatment	Decreased BWC; increased NS
Pakkianathan et al. 2016 [66]	Rat	Propofol pre-treatment	No effect on BWC or NS
Sherchan et al. 2016 [38]	Rat	Recombinant Slit2 pre-treatment	Decreased BWC, CD45 antigen, myeloperoxidase, cell division cycle protein 42 (Cdc42); increased NS
Yang et al. 2016 [42]	Rat	Allogenic myelin basic (MB) protein vs. autogenic brain cell suspension pre-treatment	Suppressed secondary inflammatory reactions
Kim et al. 2017 [44]	Rat	*Crotalus atrox* whole venom preconditioning	Decreased intraoperative hemorrhage, postoperative hematoma
Kim et al. 2017 [45]	Rat	*Crotalus helleri* whole venom preconditioning	Decreased BWC and COX-2 over-expression; increased NS
Sherchan et al. 2017 [37]	Rat	Recombinant Slit2 pre-treatment	Decreased BBB permeability; increased BBB junction proteins, NS
Wang et al. 2017 [67]	Rat	*Naja sputatrix* whole venom preconditioning	Decreased pro-inflammatory mediators; increased NS
Wang et al. 2018 [68]	Rat	PLA_2_ preconditioning	Decreased BWC, intraoperative bleeding; increased NS
Xiao et al. 2018 [69]	Rat	Milk fat globule-epidermal growth factor-8 (MFGE8) pre-treatment	Decreased BWC, apoptotic cells; increased NS
Akyol et al. 2018 [70]	Rat	Neurotrophin-3 intraoperative	Decreased BWC, BBB permeability; increased NS
Chen et al. 2019 [71]	Rat	Collagen-glycosaminoglycan (CG) matrix post-treatment	Decreased ED-1, IBA-1, MPO, TNF-α, IL-6, NF-κB; increased GMCSF, IL-10
Hsu et al. 2018 [72]	Rat	Collagen-glycosaminoglycan (GC) matrix post-treatment	Increased MMP2, MMP9
Zakhary et al. 2019 [41]	Rat	RO 61-8048 (kynurenine inhibitor) post-treatment	Decreased BWC, increased NS

### Preconditioning for SBI

While preconditioning (PC) studies have demonstrated promising neuroprotective effects for several animal models of different forms of brain injury [35, 44, 45, 67, 68], it has often been noted that clinical translation is limited since many injuries occur spontaneously. In the last few decades, the potential of PC, especially for scheduled (or elective) surgeries, has become a real focus in hopes of developing an effective therapy. PC is an approach that utilizes what would be normally damaging/toxic therapies which, when given in sub-toxic amounts, induce minimal injury while provoking the body’s innate protective response, thereby reducing possible damage from a future major insult [73]. PC therapies exist for several stroke and brain injury models. For example, hypoxic/ischemic-PC has been successful in providing neuroprotection in models of stroke [74–77].

To date, more than 30 substances or treatments have been investigated as therapeutic options in SBI (Table 1). Such treatments have been administered before surgery, after surgery, and utilized in a preconditioning manner. Investigators have been able to give insight into the pathology, cellular mechanism of action, and where areas of therapeutic focus should be for treating SBI.

Now, with evidence supporting the efficacy of PC [34, 67], the need remains to investigate a translational therapy. Because of the recent discoveries of snake venom PC and a further understanding of the mechanism driving these protective effects, we have focused our attention on specific venom protein components which we believe can provide protective effects for edema and hemorrhage.

### Venom therapies

For many centuries, mankind has utilized the deadly venoms from animals as either weaponry or medical therapies. In 326 B.C.E., Alexander the Great’s army encountered arrows dipped in Russell’s viper (*Daboia russelii*) venom in India [78]. One of the first recorded medical uses of venom was described by the Roman historian Appian in 27 B.C.E., Appian wrote about the wound that Mithradates suffered, and as he was near death, his Scythian doctor administered a small amount of steppe viper (*Vipera renardi*) venom to stop the profuse bleeding and the venom caused the blood to clot which saved his life [79].

Up until the late twentieth century, venoms from a wide range of animals were used as traditional remedies in small doses. As modern medicine advanced, investigators were able to identify compounds that could have therapeutic potential [80]. Venoms are complex mixtures of peptides, proteins, and enzymes. With successful isolation, these compounds are highly selective and can be used in a safe manner as a therapeutic [81]. Such drugs as Capoten to treat hypertension, Byetta to treat type 2 diabetes mellitus, Prialt to manage severe chronic pain, and Chlorotoxin to identify tumors in the CNS have all been developed from the venoms of snakes, snails, lizards, and scorpions [82]. The hemostatic nature of many venoms makes them a prime candidate to expand PC therapy for preventing hemorrhage [80]. Moreover, many of the proteins found in venoms cause a rapid and prolonged onset of edema in a dose-dependent manner. Proteins that elicit inflammatory mechanisms similar to those of SBI are optimal for PC. Here, we describe the therapeutic potential of a specific protein isolated from one of the four major snake venom protein families, and proteins from two additional protein families.

### *Pseudechis papuanus* venom-derived phospholipase A_2_ (PLA_2_)

The Papuan black snake, *P. papuanus*, is an elapid species endemic to Papua New Guinea. Recent proteomic analysis showed that the venom proteome is dominated by a variety of PLA_2_ isoforms, which together account for approximately 90% of the venom proteins, with the remainder including a short neurotoxic three-finger toxins (3FTx; 3.1%), PIII-snake venom metalloproteinase (SVMPs; 2.8%), cysteine-rich secretory proteins (CRISPs; 2.3%), and L-amino acid oxidase (LAAO; 1.6%) molecules [83]. Venom activities including intravascular hemolysis, pulmonary congestion and edema, anticoagulation, and death are believed to be caused primarily by the PLA_2_ isoforms. After Kim et al. [45] and Wang et al. [67, 68] demonstrated that venoms with PLA_2_ can attenuate brain edema and improve neurological outcomes, we became interested in investigating a pure PLA_2_ therapy. Because of the complex nature of the venoms these investigators used, and also the presence of immunogenic proteins in these venoms, there is a need to study a PLA_2_-rich venom such as that of *P. papuanus*. Kim et al. [44] further demonstrated that venom with hemorrhagic effects can mitigate intra- and post-operative brain hemorrhage. Because of the unique mixture of inflammatory and platelet-aggregating effects of *P. papuanus* venom, we view this venom as a prime therapy for SBI, which preliminary studies are bearing out.

### Snake venom metalloproteinases

Snake venom metalloproteinases (SVMPs) are divided into three main classes (PI, PII, and PIII) based on size and domain. SVMPs are Zn^2+^-dependent endopeptidases with activities usually related to hemorrhaging and disruption of hemostasis [84]. These metalloproteinases cleave a small number of specific zymogens in platelet aggregation and trigger the coagulation cascade. Furthermore, they are known to cause edema, inflammation, and necrosis because of their destructive action on basement membranes [85, 86]. After an ischemic event, there is an increase in matrix metalloproteinases (MMPs) both in the blood and brain, with the most studied ones being MMP-9 and MMP-2 [87]. These two proteins are thought to be responsible for the degradation of collagen IV, a major component of the basal lamina, ultimately leading to BBB disruption. SVMPs have strong structural similarities with both mammalian matrix metalloproteinase and members of a disintegrin and metalloproteinase (ADAMs) groups [88]. Studies have demonstrated that SVMPs induce formation of blisters in the dermis and infiltration of leukocytes at the site of injection. Injection of SVMPs was also associated with degranulation of mast cells (which lead to histamine release, inducing vascular permeability and vasodilatation leading to extravasation), and the expression of messenger RNA (mRNA) encoding for tumor necrosis factor (TNF), interleukin-1 (IL-1), and interleukin-6 (IL-6) by elicited macrophages [86, 89].

Of particular interest is the ability of SVMPs to activate the complement system. Activation of anaphylatoxins C3a and C5a serve as powerful chemoattractants for leukocytes and have been shown to damage the BBB in ICH [90]. Anaphylatoxins induce rapid activation of endothelial cells and resident microglia, as well as infiltration of granulocytes, in the perihematomal region [91, 92]. Activated microglia secrete inflammatory cytokines, such as TNF-α and IL-1β, thereby amplifying the inflammatory response [93]. The formation of the membrane attack complex (MAC) causes the lysis of erythrocytes and exacerbates brain edema and oxidative stress due in part to the breakdown of hemoglobin [94, 95]. Collectively, SVMPs can trigger similar inflammatory pathways that have been implicated in SBI, causing an endogenous response to protect against future insult.

### Snake venom serine proteases

Snake venom serine proteases (SVSPs) comprise a group of well-studied toxins which are known for being the primary contributor affecting the hemostatic system [96]. Serine proteases are abundant in snake venoms and have been identified in venoms mainly from the subfamilies Crotalinae (genera *Agkistrodon*, *Crotalus*, *Lachesis*, *Trimeresurus*), Viperinae (*Cerastes cerastes*, *Cerastes vipera*, *Bitis gabonica*), and Colubrinae (*Dipholidustypus*) [96]. SVSPs cause interference and imbalances of the hemostatic system by promoting specific proteolysis at various key points of the coagulation cascade [97, 98]. Furthermore, SVSPs have been demonstrated to induce significant edema via the metabolism of arachidonic acid (AA), involving protease-activated receptors (PARs), protein kinase c (PKC), phospholipase C (PLC), and cyclooxygenase-2 (COX-2) receptors, and also induce a significant increase in malondialdehyde (MDA) levels [99]. Costa et al. [99] demonstrated that SVSPs may be involved in the degradation of PAR1 and PAR2, which activate PLC and PKC to mobilize AA, while increasing oxidative stress. SVSPs trigger inflammatory cascades that have been implicated in SBI pathophysiology that SVMPs and PLA_2_ might not successfully trigger. Using SVPSs as a preconditioning agent may also attenuate the injury due to SBI.

## Conclusions

The anticipatable timing of surgical brain injury provides a unique opportunity for preemptive intervention, but clinical medicine has yet to utilize preconditioning methods to protect the brain from SBI. To date, SBI is often left to resolve on its own, and currently, there is no treatment available to alleviate it, which is in large part due to our poor understanding of the pathophysiology.

Before any of these therapies can be tested in clinical trials, further in vivo experimental studies are needed to evaluate preconditioning agents and to provide a better mechanistic understanding of SBI pathophysiology. The pathophysiological understanding of SBI remains sparse compared to that of other stroke or brain injury models. To date, SBI studies have implicated certain pro-inflammatory pathways and cellular targets. Further studies are needed to expand on upstream and downstream mediators of these signaling pathways in the pathogenesis of SBI. Secondary injury processes of SBI include neuroinflammation, metabolic disturbances, apoptosis, ischemia, oxidative stress, and BBB disruption. Moreover, studies are needed with isolated snake venom metalloproteinase and snake venom serine proteases. Whole venoms have been investigated in SBI models and have been efficacious in small quantities. Purified proteins have been extensively studied, and their properties suggest they could provide further neuroprotection. Despite their toxic effects, it is well established that some components from snake venoms present beneficial effects when acting alone in small quantities.

Lastly, studies that factor in sex and age are also needed. In TBI, it is believed that sex plays a role in outcomes and response to TBI treatments. Microglia, which are the major resident immune cells of the brain, have sexually dimorphic roles in the development and maintenance of the normal brain and have different responses in TBI between males and females [100]. Age at the time of injury is a major factor in the functional recovery of patients. Investigators demonstrated that there is an increase in infiltration of peripheral monocytes at the site of injury in aged rates compared to young animals in a TBI model [101]. As we continue to investigate SBI and its pathophysiology, there is hope that additional therapeutic targets may arise. Venom-derived proteins applied in a preconditioning manner [102, 103] is a promising translational therapy and could be a major step forward in how we treat patients.

## Data Availability

Not applicable.

## Abbreviations

AA: Arachidonic acid
ADAM: A disintegrin and metalloproteinase
AQ-4: Aquaporin-4
BBB: Blood-brain barrier
BWC: Brain water content
CDc42: Cell division cycle protein 42
CRISPs: Cysteine-rich secretory proteins
CNS: Central nervous system
CSF: Cerebral spinal fluid
COX-2: Cyclooxygenase-2
FGF2: Fibroblast growth factor 2
GM-CSF: Granulocyte-macrophage colony-stimulating factor
GSH: Glutathione
HIF1A: Hypoxia-inducible factor 1-α
IBA-1: Ionized calcium binding adaptor molecule 1
ICP: Intracranial pressure
IL-1/6/10: Interleukin-1/6/10
KO: Knockout
LAAO: L-amino acid oxidase
LPO: Lipid peroxidation
MAC: Membrane attack complex
MBP: Myelin basic protein
MDA: Malondialdehyde
MMP: Matrix metalloproteinase
MPO: Myeloperoxidase
mRNA: messenger RNA
NF-κB: Nuclear factor kappa light chain enhancer of activated B
NO: Nitric oxide
NS: Neurological score
PARs: Protease-activated receptors
PC: Preconditioning
PDGF: Platelet derived growth factor
PKC: Protein kinase c
PLA_2_: Phospholipase A_2_
PLC: Phospholipase C
SBI: Surgical brain injury
SVMPs: Snake venom metalloproteinase
SVSPs: Snake venom serine protease
TGF-β1: Transforming growth factor beta 1
TBI: Traumatic brain injury
TNF: Tumor necrosis factor
3FTx: Three-finger toxin
VEGF: Vascular endothelial growth factor
ZO-1: Zonula occludens-1
